# Aryl hydrocarbon receptor: current perspectives on key signaling partners and immunoregulatory role in inflammatory diseases

**DOI:** 10.3389/fimmu.2024.1421346

**Published:** 2024-08-15

**Authors:** Fatemah Bahman, Khubaib Choudhry, Fatema Al-Rashed, Fahd Al-Mulla, Sardar Sindhu, Rasheed Ahmad

**Affiliations:** ^1^ Department of Immunology & Microbiology, Dasman Diabetes Institute, Dasman, Kuwait; ^2^ Department of Human Biology, University of Toronto, Toronto, ON, Canada; ^3^ Department of Translational Research, Dasman Diabetes Institute, Dasman, Kuwait; ^4^ Animal & Imaging Core Facilities, Dasman Diabetes Institute, Dasman, Kuwait

**Keywords:** Aryl hydrocarbon receptor, AhR, immune regulation, signaling pathways, inflammatory diseases

## Abstract

The aryl hydrocarbon receptor (AhR) is a versatile environmental sensor and transcription factor found throughout the body, responding to a wide range of small molecules originating from the environment, our diets, host microbiomes, and internal metabolic processes. Increasing evidence highlights AhR’s role as a critical regulator of numerous biological functions, such as cellular differentiation, immune response, metabolism, and even tumor formation. Typically located in the cytoplasm, AhR moves to the nucleus upon activation by an agonist where it partners with either the aryl hydrocarbon receptor nuclear translocator (ARNT) or hypoxia-inducible factor 1β (HIF-1β). This complex then interacts with xenobiotic response elements (XREs) to control the expression of key genes. AhR is notably present in various crucial immune cells, and recent research underscores its significant impact on both innate and adaptive immunity. This review delves into the latest insights on AhR’s structure, activating ligands, and its multifaceted roles. We explore the sophisticated molecular pathways through which AhR influences immune and lymphoid cells, emphasizing its emerging importance in managing inflammatory diseases. Furthermore, we discuss the exciting potential of developing targeted therapies that modulate AhR activity, opening new avenues for medical intervention in immune-related conditions.

## Introduction

The Aryl hydrocarbon receptor (AhR) is a ubiquitously-expressed, ligand-activated transcription factor that belongs to the basic helix-loop-helix/per-arnt-sim (bHLH/PAS) superfamily of the sensors of foreign and endogenous signals or ligands and it was initially identified for its involvement in metabolizing xenobiotics, particularly those containing aromatic hydrocarbons (1). In 1976, Poland et al. demonstrated the strong binding of 2,3,7,8-tetrachlorodibenzo-p-dioxin (TCDD - a contaminant that belongs to chemical herbicide Agent Orange) to a cellular component in mouse liver cells, a significant finding that shed light on how the liver absorbs this compound (2). Further investigation has revealed that AhR plays essential roles in maintaining homeostasis, with its domain structures and functions being conserved across evolution and commonly observed in nearly all multicellular organisms (3, 4). Upon ligand binding to AhR in the cytoplasm, it translocates to the nucleus and forms a complex with AhR nuclear translocator (ARNT) (5). This complex acts as a transcription factor, binding to xenobiotic responsive elements (XRE) and regulating the expression of many prototypic genes belonging to the cytochrome P450 family, including CYP1A1, CYP1A2, and CYP1B1 (6). As opposed to other bHLH/PAS proteins, AhR is the only member of this superfamily that is known to bind naturally occurring xenobiotics (3). Mutations in the AhR gene or near AhR target genes are associated with human diseases, primarily through mechanisms involving altered xenobiotic metabolism, disrupted gene regulation, and impaired immune function (7–14).

Recent research studies spanning over the past two decades have identified numerous endogenous ligands of AhR, revealing various pathophysiological functions of the receptor beyond its initially-investigated toxicological aspects (15). Moreover, numerous *in vivo* studies have underscored the importance of AhR in normal development, linking AhR deficiency to conditions such as cardiac hypertrophy, epidermal hyperplasia, and other abnormalities (16, 17). This focused review aims to outline significant advancements in understanding how AhR regulates physiological functions, with emphasis on its roles in the immune system.

## Structure of AhR

**Figure 1:**
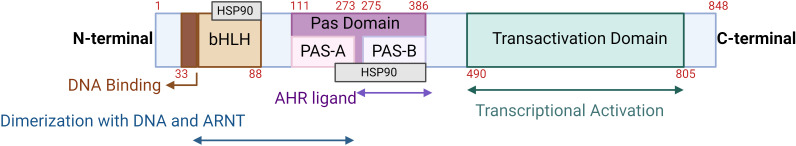
Schematic representation of the human AhR structure: The three different domains include the N-terminal bHLH domain, Per-ARNT-Sim (PAS) domains (PAS A and PAS B), and a C-terminal transactivation domain. The numbers in red represent the amino acids spanning each domain. This illustration was created with Biorender.com.

AhR has three different domains, namely an N-terminal bHLH domain, Per-ARNT-Sim (PAS) domains (A and B), and a C-terminal transactivation domain (TAD), as shown in **Figure 1** (18). While the three-dimensional structure of AhR remains elusive, the crystal structure of the AhR-ARNT-XRE complex has been elucidated (19). Analysis of this AhR-ARNT-XRE complex suggests that the stability of AhR-ARNT heterodimerization and the interaction of AhR interdomains are basically regulated by the bHLH and PAS domains (20). The AhR activation pathway includes ligand binding, nuclear translocation, and finally binding to the canonical XRE of target genes (21). The PAS-B domain containing a conserved ligand-binding pocket detects the xenobiotic signals (22). Mutation studies have led to the identification of key residues in mice, such as Ala375, His285, and Gln377, that play crucial roles in ligand binding (23). Contrary to PAS-B, the PAS-A domain mainly regulates the specificity and stability of heterodimerization with ARNT. Further studies reveal that the N-terminal α-helical structure forms an integral dimer interface together with hydrophobic interactions with residues in the partnering PAS-A domain to ensure stability between AhR and ARNT (24). The bHLH domain is particularly involved in the identification of the XRE consensus sequence, TTGCGTG, via the two N-terminal α-helices and a flexible connector (19, 25).

## AhR activation and crosstalk with key signaling pathways

### AhR activation

**Figure 2:**
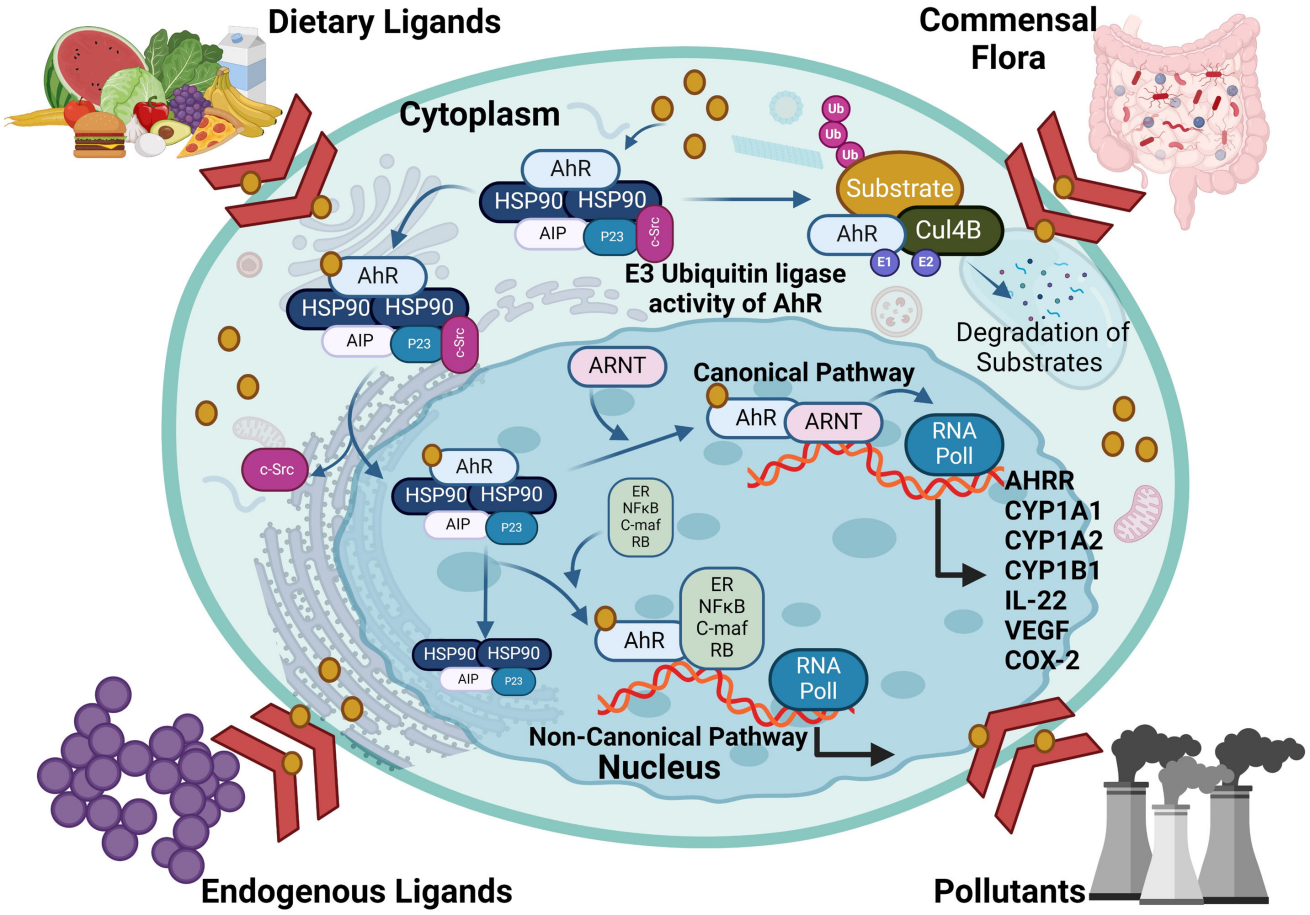
The activation pathways of AhR: In the genomic (canonical) pathway, an inactive form of the AhR is cytoplasmic and complexed with HSP90, AIP and SRC. Upon ligand binding, the AhR complex translocates to the nucleus, where the AhR forms a complex with ARNT and binds to xenobiotic response element, inducing AhR-target gene expression. The AhR non-canonical pathway also induce transcription of genes involved in inflammation, immune response and/or development. AHRR competes with the AhR for binding with ARNT and forms the inactive heterodimer AHRR-ARNT. The dissociation of the AhR transcriptional complex leads to translocation of the AhR to the cytoplasm, where it is degraded via the proteasomal pathway. AhR, aryl hydrocarbon receptor; AHRR, AhR repressor; ARNT, AhR nuclear translocator; AIP, AhR-interacting protein and Ub, ubiquitin. This illustration was created with Biorender.com.

As a member of the PAS (Per-ARNT-Sim) family, AhR operates as a transcription factor, with its PAS protein domain characterized by a fundamental spiral ring helix structure, enabling diverse responses to various environmental pollutants and cellular metabolites (19). In an inactive state, AhR resides in the cytoplasm and forms a complex with stabilizing chaperones. This cytoplasmic AhR complex consists of several components including (A) a dimer of heat shock protein 90 (HSP90) which helps maintain AhR in a conformation essential for the optimum ligand affinity (26), (B) AhR-interacting protein (AIP) which potentiates stability of the AhR-HSP90 complex (27), (C) a 23-kDa glycoprotein (p23) which acts as a co-chaperone and shields the AhR from ubiquitin-mediated degradation, as does the AIP, and ensures the cytoplasmic localization (28), and (D) c-Src which participates in the early stages of AhR activation following binding with ligand, contributing to non-genomic (non-canonical) aspects of AhR-mediated signaling (29). In the AhR genomic (canonical) pathway, after ligand binding with the AhR complex in the cytoplasm, a conformational change occurs, leading to the dissociation of the complex and nuclear translocation. Within the nucleus, its binding partner ARNT, also called hypoxia-inducible factor 1β (HIF-1β), orchestrates the gene transcription after binding with dioxin response elements (DREs) at the AhR-ARNT binding sites (30, 31). The activation pathways of the AhR are illustrated in **Figure 2**.

**Figure 3:**
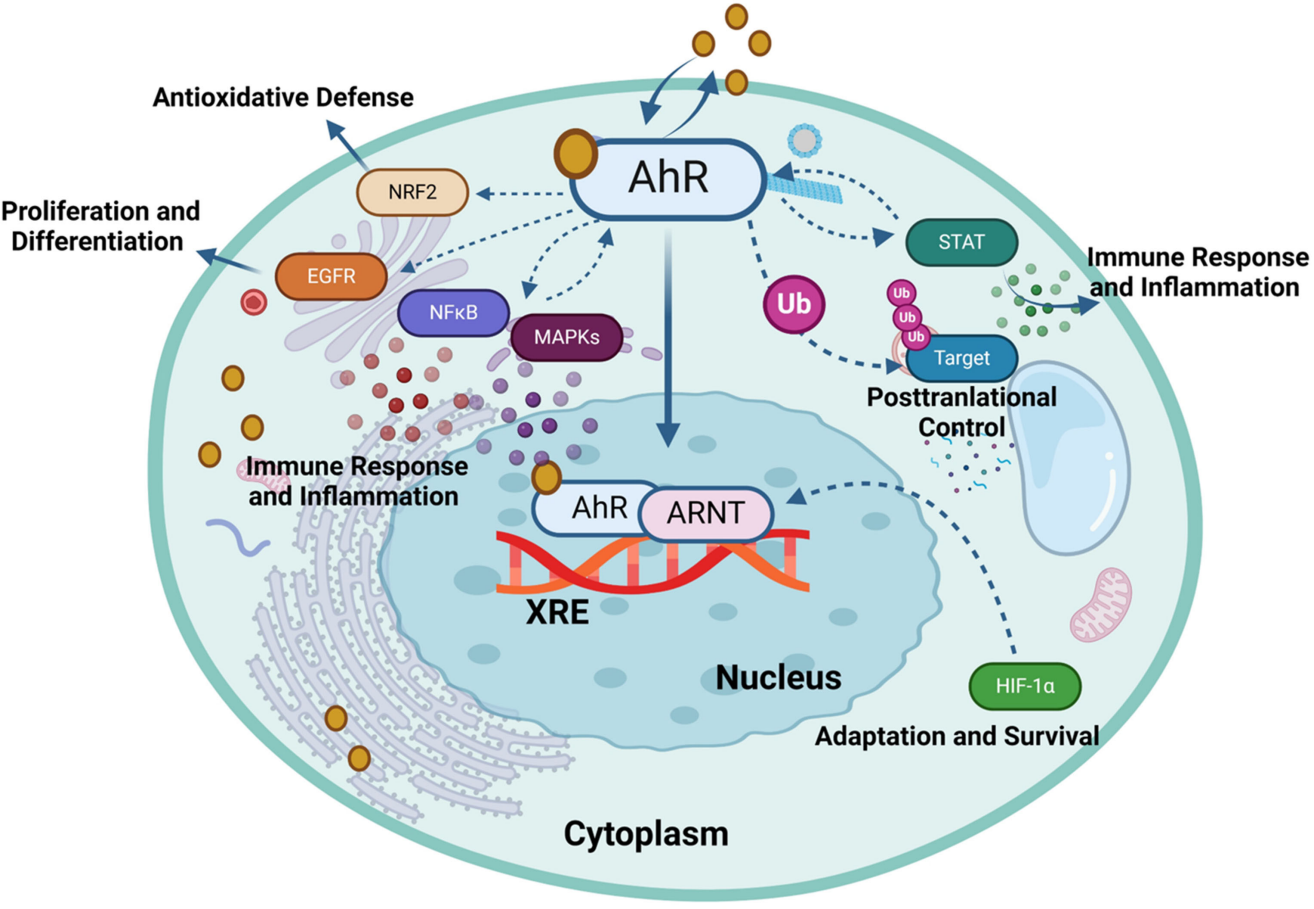
Aryl hydrocarbon receptor (AhR) regulates gene expression in response to environmental and endogenous stimuli. The activation of the AhR can modulate several signaling pathways, each contributing to diverse biological functions such as HIF-1α, NF-κB, Nrf2, MAPK, EGFR, JAK/STAT and ubiquitin-proteasome (Ub) pathways. This illustration was created with Biorender.com.

A negative feedback mechanism regulates the AhR activation by degrading AhR ligands through cytochrome P450 (CYP1) enzymes such as CYP1A1 and CYP1A2. Additionally, the AhR repressor (AhRR) and the AhR ligand complex compete each other for forming a heterodimer with ARNT, thereby repressing AhR-dependent transcription (32). Notably, AhRR is structurally similar to the AhR, however, it cannot bind with ligands due to lacking its N-terminal PAS B domain (33). Similarly, HIF-1α also competes ARNT for interaction with AhR, a process that influences the degradation of HIF-1α and mediates the differentiation of type 1 regulatory T (Tr1) cells (34). AhR governs diverse biological responses by interacting with exogenous and endogenous ligands, leading to increased transcription of phase I xenobiotic metabolizing enzymes, such as CYP1A1, CYP1B1, tryptophan 2,3-dioxygenase (TDO2), and indoleamine 2,3‐dioxygenase 1 (IDO1). AhR is involved in the transcriptional regulation of diverse genes, from cytokines including IL-10, IL-17, and IL-22, to ectonucleotidases such as CD39 and CD73, as well as the ATP-binding cassette (ABC) family of drug transporters (35). AhR expression in Th17 cells promotes the production of IL-17A,F and IL-22 (36, 37). The dynamics between ILC3 cells and IL-22 expression require the AhR involvement, and it was found that the absence of AhR leads to a reduction in IL-22-producing ILC3 cells (38). Notably, CD39 (also known as ectonucleoside triphosphate diphosphohydrolase-1 or ENTPD1) contributes to immunosuppression mainly via its role in the adenosinergic signaling pathway (39). Briefly, the extracellular ATP/ADP molecules released by damaged or stressed cells may act as proinflammatory signals, are hydrolyzed into AMP by CD39 ectonucleotidase. Another ectoenzyme called CD73 (ecto-5’-nucleotidase) converts the AMP into adenosine which is a potent immunosuppressive molecule and can bind to its receptors on important immune effector cells such as T-cells, macrophages, natural killer (NK) cells, and dendritic cells (DCs). During T-cell priming, CD39 inhibits the costimulatory ATP signals and promotes the adenosine-mediated immunosuppression (40). Adenosine binding to its A2A receptor on T-cells can lead to (1): inhibition of T-cell activation and proliferation (2); suppression of proinflammatory cytokines’ production (3); induction of anti-inflammatory cytokines (4); inhibition of NK cell cytotoxicity; and (5) induction of immunosuppressive regulatory T-cells (Tregs) (41). Thus, CD39 contributes to adenosine-mediated immunosuppression via the CD39-CD73-adenosine-A2AR pathway (42). Recent investigations indicate that AhR binding to the endogenous ligand unconjugated bilirubin (UCB) leads to CD39 upregulation in Th17 cells, conferring upon Th17 cells substantive immunosuppressive properties (43). Conversely, under hypoxia from protracted inflammation, the AhR ligation by UCB in pathogenic Th17 cells from Crohn’s disease patients results in HIF-1α-dependent CD39 downregulation and defective immunosuppression in response to UCB due to resistance of Th17 cells to AhR signaling and induction of ATP-binding cassette (ABC) transporters () (35). Tregs are a specialized subset of CD4+ T cells that are crucial for maintaining immune homeostasis and self-tolerance. Treg cells produce immunosuppressive cytokines such as IL-10, TGF-β, and IL-35 (44). These cytokines inhibit the proliferation and function of effector T cells, dampening the immune response and reducing inflammation resulting in preventing excessive immune responses that could lead to autoimmune diseases. Treg cells are integral to the adaptive immune system, characterized by its ability to recognize specific antigens and generate a tailored immune response (45). Moreover, Tregs cells express CTLA-4 (Cytotoxic T-Lymphocyte Antigen 4), which binds to CD80/CD86 on antigen-presenting cells (APCs) (46). This interaction reduces the APCs’ ability to provide the necessary co-stimulatory signals for T effector cells activation. Treg cells can directly interact with B cells, suppressing their proliferation and differentiation into plasma cells, which are responsible for antibody production (47). Their ability to suppress effector T cells and regulate B cell responses highlights their crucial role in immune homeostasis. While type 1 Tregs (Tr1 cells) are a subtype of regulatory T cells which are pivotal in maintaining peripheral immunity by regulating tolerance towards a wide range of antigens. Tr1 cells play a central role in maintaining immune homeostasis and are highly instrumental in preventing the T cell-mediated diseases including autoimmunity, allograft rejection, allergies, Graft-versus-host disease (GvHD), and chronic inflammatory diseases (48). The transcription biomarkers of Tr1 cells include the AhR, interferon regulatory factor (IRF)-4, repressor of GATA-3 (ROG), early growth response protein (Egr)-2, and musculoaponeurotic fibrosarcoma (c-Maf; a cellular homolog of a viral oncogene) (48). Tr1 cells express increased levels of IL-10 and lack in constitutive expression of the forkhead box P3 (Foxp3) (49). Other cytokines expressed by Tr1 cells include the TGF-β, IFN-γ, and IL-5 but no IL-2, IL-4, and IL-17 (50). Tr1 cells act as key regulators in immune network and mediated immune suppression and tolerance through multiple mechanisms including cytokine expression (especially, IL-10 and TGF-β), cell to cell contact (via inhibitory receptors CTLA-4 and PD-1), metabolic disruption (by expressing CD39 and CD 73 ectoenzymes that produce adenosine and increase the intracellular cAMP levels), and cytolytic activity (through expression of granzymes A/B and perforin) (51). AhR regulates the expression of IL-10 and IL-21 in Tr1 cells (52, 53). Interestingly, the suppression of CD39 expression was observed in Tregs and Th17 cells from individuals with autoimmune hepatitis. This dysfunction in immunosuppressive conditions was related to dysregulated AhR signaling and interventions targeting the dysregulated AhR pathway restored CD39 upregulation in Tregs and Th17 cells (54). AhR has also been linked to the viral pathogenic response. Specifically, ocular infection with herpes simplex virus can lead to a chronic immune inflammatory reaction that may result in blindness. However, in a murine model, a single dose of TCDD was found to alleviate herpes keratitis lesions, reduce viral load, and decrease levels of pro-inflammatory cytokines. Of note, FICZ did not exhibit the same efficacy, highlighting differences between these two AhR ligands (55). Taken together, AhR is a ligand-activated transcription factor that translocates to the nucleus upon formation of a heterodimer complex with ARNT and subsequently binds to XRE sequences, thereby regulating gene expression of multiple biochemical pathways, each contributing to a wide range of biological processes, as illustrated in **Figure 3** (56).

In the absence of a ligand, AhR remains sequestered in the cytoplasm together with chaperones and immunophilin-like proteins, such as co-chaperone p23, c-Src tyrosine kinase, HSP90, and AIP. Upon binding with an agonist, conformational changes occur in AhR, prompting its translocation to the nucleus where it engages with ARNT and the resultant heterodimer complex then binds to a specific XRE and coregulators in the promoter region of AhR target genes to regulate their transcription. AhR facilitates the expression of CYP enzymes that degrade the AhR ligands. After its export from the nucleus, AhR is rapidly eliminated by proteasomal degradation in the cytoplasmic compartment, following its covalent binding with ubiquitin, whereas, SUMOylation favors the AhR stability by inhibiting ubiquitination (57). Besides, as a part of the negative regulatory loop mechanism, AhR triggers the expression of its own repressor AhRR, which precludes the formation of the AhR/ARNT complex required for AhR-mediated signaling. Proteasomal degradation of AhR and negative regulatory loop ensure a temporal control of overstimulation by AhR agonists. In the following section, we review the crosstalk between AhR/ARNT and other signaling partners or pathways in inflammatory conditions.

### Interaction between AhR and hypoxia inducible factor-1 alpha

Hypoxia-inducible factor 1-alpha (HIF-1α) is a member of the class I bHLH/PAS protein superfamily and it functions as a critical oxygen sensor and transcriptional regulator of the balance or oxygen homeostasis between metabolic demand and vascular oxygen supply through the increased angiogenesis (58). Unlike ARNT, which is ubiquitously expressed, the HIF-1α expression is based on intracellular oxygen levels, and under normal oxygen conditions, HIF-1α is rapidly targeted for ubiquitination and proteasomal degradation (59). In hypoxic conditions, HIF-1α is stabilized or protected from proteasomal degradation, allowing it to translocate to the nucleus and form heterodimer complexes with ARNT, orchestrating expression of a multitude of hypoxia-responsive genes containing the hypoxia-response element or a 5′-G/ACGTG-3′ motif, such as carbonic anhydrase-IX (*CAIX*) and vascular endothelial growth factor (*VEGF*) (60, 61). Given that ARNT is a dimerization partner of both the HIF-1α and AhR, crosstalk between these two signaling pathways is not unexpected. Additionally, the binding of AhR to ARNT becomes indispensable for an AhR-driven immune response, with HIF-1α intricately regulating interference in AhR/ARNT transcriptional activity. Tr1 cells, identified as Foxp3^–^ regulatory CD4^+^ T cells, pose challenges in understanding their differentiation and metabolic control. Increasing evidence suggests that aerobic glycolysis plays a pivotal role in enhancing Tr1 cell differentiation through metabolic programs controlled by both HIF-1α and AhR (34, 62). It is noteworthy that HIF-1α oversees early metabolic reprogramming in Tr1 cells, and subsequently, AhR facilitates HIF-1α degradation, regulating Tr1 cellular metabolism. Thus, the AhR and HIF-1α mutually collaborate in sustaining Tr1 cellular metabolism, thereby contributing to modulation of the immune response of Tr1 cells (34). Norisoboldine (NOR), a recently-identified natural AhR ligand, shows a great potential in reducing osteoclast differentiation and inflammatory bone erosion (63). NOR achieves this by activating AhR and subsequently inhibiting HIF-1α signaling pathways. Mechanistically, NOR securely binds to AhR, enhances AhR’s movement into the nucleus, increases accumulation of the AhR-ARNT complex, and thus impedes accumulation of the ARNT-HIF-1α complex in RAW 264.7 cells (64). Furthermore, NOR facilitates the differentiation of Tregs in a mouse colitis model under hypoxic conditions, thus alleviating the onset of colitis (63). In Crohn’s disease, Th17 cells isolated from the peripheral circulation were found to have reduced CD39 expression, which played a role in their non-responsiveness to immunosuppressive effects of unconjugated bilirubin (UCB), which is an endogenous AhR ligand (35). Thus, chronic inflammation-induced hypoxia leads to an increase in ABC transporters and HIF-1α expression which significantly impairs the AhR/ARNT signaling due to diminished availability of AhR ligands. Besides, the elevated ABC transporters activity causes efflux of AhR ligands, such as UCB, from Th17 cells and further reduces the AhR substrate availability. Not surprisingly, this defect in Th17 cell responsiveness to AhR stimulation by UCB was effectively restored when HIF-1α or ABC transporter activity was inhibited (35). Altogether, the complex interaction between AhR/ARNT and HIF-1α signaling axes leads to immune cell differentiation reprogramming and aggravated pathogenesis of certain autoimmune diseases.

### Interaction between AhR and nuclear factor-κB pathway

NF-κB, an important regulator of both the innate and adaptive immune responses, is intricately linked to various signaling pathways. The complex interplay between NF-κB and AhR in different cell types and disease states is an attractive subject of ongoing research (65). Emerging evidence supports the role of 3,3′-Diindolylmethane (DIM), an active metabolite of cruciferous indole-3-carbinol (I3C), in the regulation of the AhR-mediated cellular immune responses. DIM was found to reverse epithelial-mesenchymal transition (EMT) and prevent cancer cell metastasis by modulating AhR signaling and suppressing the NF-κB pathway (66). Indeed, kynurenine (KYN)-induced AhR signaling in gliomas was found to affect macrophage polarization and phenotype and the AhR depletion led to increased NF-κB activation *in vitro*. Mechanistically, AhR null mice showed upregulated SOCS2 expression and degradation of Krüppel-like factor 4 (KLF4) (67). Notably, the regulatory role of NF-κB extends to the expression of KYN pathway genes and AhR, which is an endogenous KYN receptor, in triple-negative breast cancer (TNBC), suggesting that NF-κB activity positively regulates the expression of key genes related to tryptophan catabolism, making inhibitors of tryptophan 2,3-dioxygenase (TDO2) an attractive therapy for treating TNBC (68). In conclusion, the AhR and NF-κB signaling pathways regulate each other, resulting in cell type- and stimulus-specific inflammatory responses.

### Interaction between AhR and nuclear factor-erythroid factor 2-related factor 2 pathway

**Figure 4:**
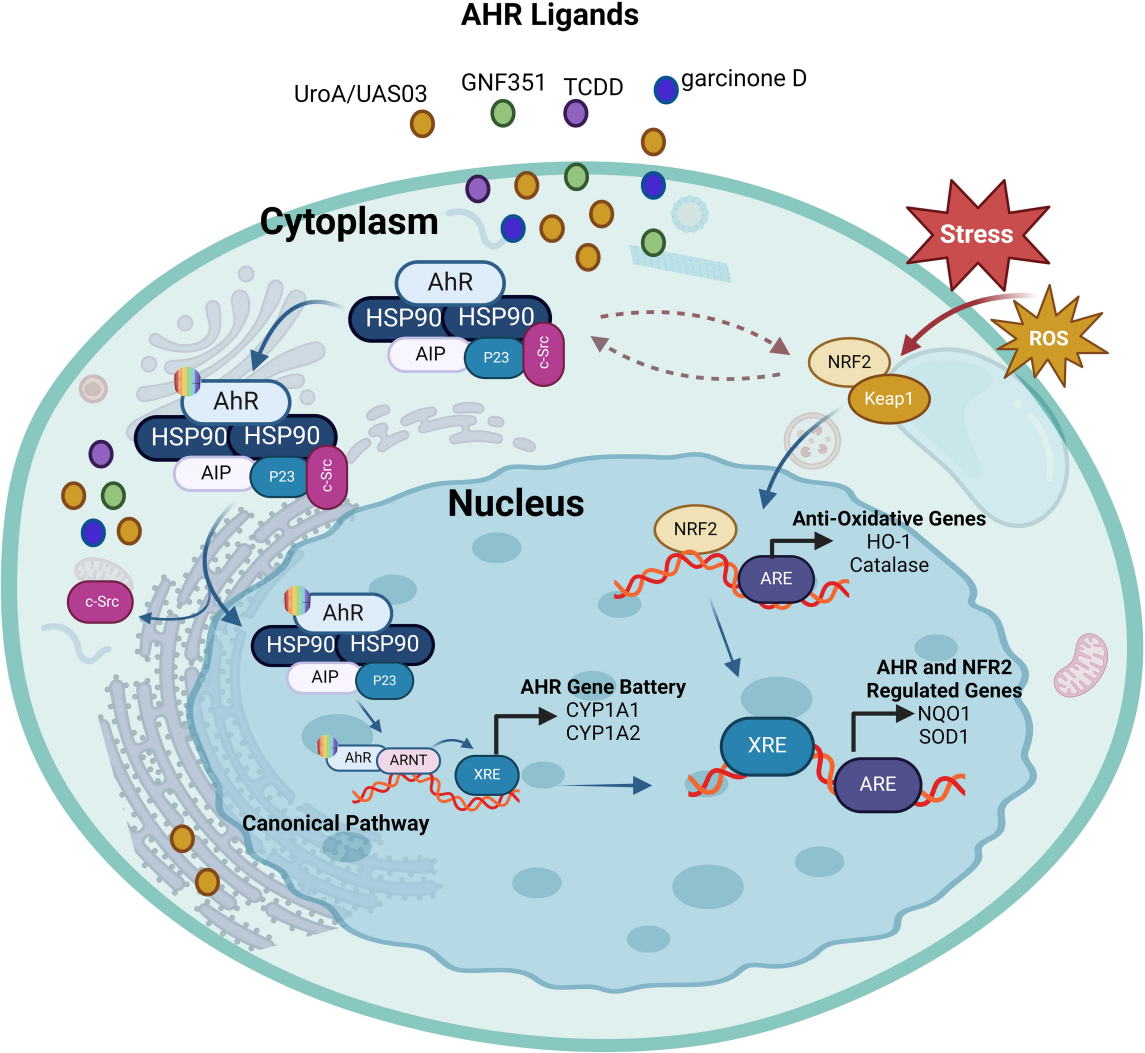
The interplay between AhR and Nrf2. Ligand-activation of AhR results in its nuclear translocation, where it dimerizes with ARNT and induces the transcription of xenobiotic-responsive element (XRE)-regulated phase I and II detoxifying enzymes, and Nrf2. Phase I detoxifying enzymes convert AhR ligands into reactive metabolites which again can lead to the formation of reactive oxygen species (ROS). ROS can trigger the dissociation of the cytosolic Nrf2-KEAP1 complex resulting in the nuclear translocation of Nrf2. This results in the expression of antioxidative response elements (ARE)-controlled phase II detoxifying enzymes, as well as the AhR. Furthermore, there is some overlap between the AhR and Nrf2 target gene batteries; for example, both routes regulate the expression of the genes NQO1 and SOD1. This illustration was created with Biorender.com.

As a regulator responding to planar aryl hydrocarbons, AhR signaling plays a key role in xenometabolism. Similarly, the Kelch-like ECH-associated protein 1 (KEAP1)-Nrf2 system, a crucial anti-oxidant defense mechanism in living systems, also plays key role in xenobiotic metabolism. KEAP1 is a sensor protein detecting contaminants to activate the transcription factor Nrf2 which regulates expression of anti-oxidant response element (ARE)-containing enzymes that orchestrate anti-oxidant defense, such as catalase (CAT), superoxide dismutase (SOD), glutathione reductase (GR), glutathione peroxidase (GPx), heme oxygenase 1 (HO-1), and peroxiredoxin (PRX), etc. (69). Nrf2 also promotes the expression of enzymes that are involved in detoxification or protection from immune toxicity including the nicotinamide adenine dinucleotide phosphate (NADPH) dehydrogenase (quinone 1/NQO1), phase III transporters, and glutathione-S-transferase (GST), among others (70). There is a link between the AhR and the glutathione cycle. AhR is known to regulate the expression of detoxification pathways enzymes involved in phase I (cytochromes P450 enzymes) and phase II (conjugation enzymes such as glutathione S-transferases). Glutathione (GSH) plays a pivotal role in cellular anti-oxidant defense and detoxification of xenobiotics and endogenous compounds. AhR activation enhances the expression of enzymes involved in glutathione synthesis and utilization (71). Thus, the AhR pathway connects with the glutathione cycle by regulating enzymes that are involved in glutathione metabolism, resulting in the increased detoxification of xenobiotics, and improved cellular antioxidant defense. The interplay between AhR and Nrf2, AhR can induce Nrf2 via several mechanisms. AhR activation can lead to upregulation of Nrf2-dependent genes that are involved in antioxidant defense and detoxification pathways (72). Notably, the Nrf2 gene promoter contains at least one functional xenobiotic response element (73). The AhR gene promoter also has several anti-oxidant response elements (AREs) (74). Nrf2 was found to regulate the expression of AhR and modulate several downstream events of the AHR signaling cascade including (1): transcriptional regulation of the xenobiotic metabolism genes (Cyp1a1 & Cyp1b1) and (2) adipogenesis inhibition in mouse embryonic fibroblasts (MEFs) (75). Thus, Nrf2 directly modulates AhR signaling, highlighting the bidirectional interactions of these pathways of cellular stress and metabolism (75). The crosstalk studies between AhR and Nrf2 mainly focused on their regulatory roles in xenobiotic metabolizing enzymes. In one such study, Ma et al. showed that Nrf2 was involved in the induction of NQO1 by TCDD, providing a new insight into the mechanism that deciphers how Nrf2 regulates phase II enzymes’ induction through the AhR ligands and phenolic antioxidants (76). It is known that skin exposure to ultraviolet B (UVB) may lead to damage through mechanisms involving oxidant stress, DNA damage, and apoptosis. Gao et al. demonstrated that activation of keratinocyte growth factor (KGF)-2 had a photoprotective effect against UVB exposure-mediated skin damage by ameliorating oxidative stress, DNA damage, mitochondrial dysfunction, and apoptosis as well as AhR/Nrf2 signaling, while this protective effect was significantly blocked by the AhR antagonist GNF351. This suggests that by promoting AhR/Nrf2 signaling, KGF-2 plays the role of an antioxidant, following UVB irradiation (77). Urolithins, such as UroA and UroB, are the natural polyphenol ellagic acid metabolites produced by gut microbiota and are known to have anti-inflammatory and anti-cancer effects (78). In a study unraveling dual beneficial effects of the microbial metabolite urolithin A (UroA) and its synthetic analog (UAS03) in colonic disorders, Singh et al. demonstrated that activation of the AhR/Nrf2 signaling cascade exerted anti-inflammatory effects and improved the intestinal barrier function by promoting expression of tight junction (TJ) proteins (79). The indispensability of AhR/Nrf2 signaling was further validated, showing that UroA/UAS03 activation did not induce expression of gut epithelial tight junction proteins and protect from experimentally-induced colitis in AhR- and Nrf2-KO mice (80). An *in vitro* study using HT-29 cell model also corroborated that xanthones, such as garcinone D, inhibited reactive oxygen species (ROS) production and improved epithelial barrier function and TJ expression by promoting the AhR/Nrf2 mediated signaling (81). Further, in context of mucosal immunity and inflammation, Th17 cells-derived cytokines including IL-17A and IL-22 are known to play central roles and it was shown that Nrf2 activator CDDO-Im promoted the expression of IL-17A and IL-22 in CD4+T cells via the AhR-dependent mechanism which was abrogated by AhR antagonist (CH-223191) as well as in CD4-specific *Ahr* KO mice (82). Together, these studies underscore the antioxidant, anti-inflammatory, and immune modulatory effects of AhR signaling, directly regulating Nrf2 transcription, with potential therapeutic implications for inflammatory diseases using AhR agonists and inhibitors. The interaction between AhR and Nrf2 is illustrated in **Figure 4**.

### Interaction between AhR and mitogen-activated protein kinase pathway

**Figure 5:**
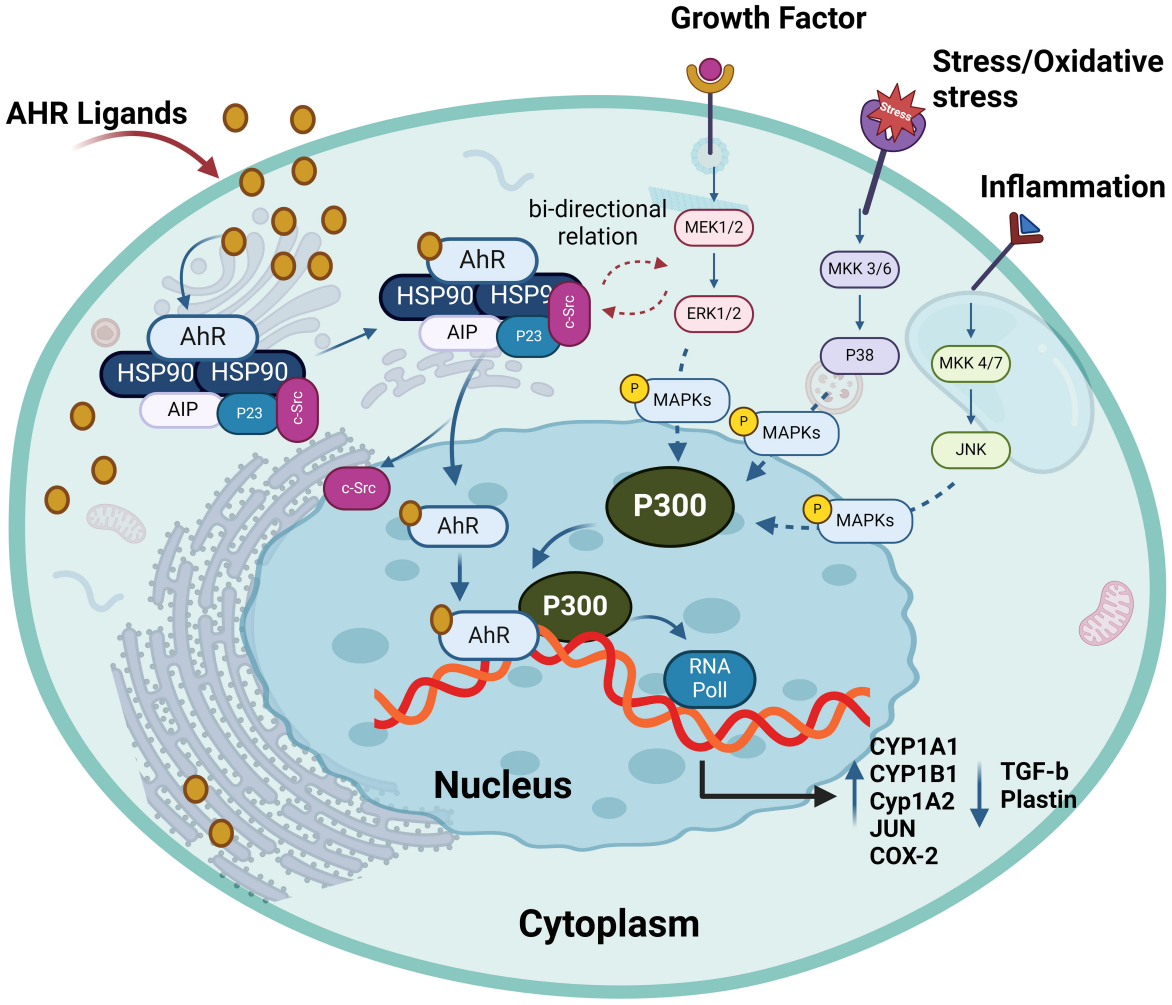
The interplay between AhR and MAPKs. AhR activation has bi-directional crosstalk between MAPKs and AhR pathways. It enhances the expression and phosphorylation of ERK1/2, P38 and MKK4/7. AhR ligands can activate one or more MAPKs, depending on the ligands and different cells leading to target gene transactivation such as enhance production of CYP1A1, CYP1A2, CYP1B1, JUN and COX-2. This illustration was created with Biorender.com.

MAPKs are serine/threonine protein kinases which coordinately regulate diverse cellular functions by transducing extracellular signals to intracellular responses, such as gene expression, metabolism, motility, mitosis, differentiation, proliferation, survival, and apoptosis. MAPKs such as p38 kinases, c-Jun N-terminal kinase/stress-activated protein kinase (JNK/SAPK), and the extracellular signal-regulated kinase 1/2 (ERK1/2) are the critical mediators of intracellular signal transduction. In general, the p38 kinases are involved in cell cycle, inflammation, and apoptosis; JNK/SAPK play roles in cellular signaling, stress-associated and immune responses, apoptosis, and in pathogenesis of metabolic disorders; while the ERK1/2 isoforms are implicated in regulation of developmental and mitogenic processes (83). Despite evidence of a cross-talk and signaling switches existing between the MAPK and AhR pathways, specific interactions between these two critical regulatory cascades remain unclear. A connection between the AhR pathway and MAPK signaling was revealed in the nervous system as the TCDD-mediated AhR stimulation of rat pheochromocytoma PC12 cells enhanced the NGF-induced neurofilament light (NFL) expression as well as ERK1/2 and p38 phosphorylation. Inhibition of MAPK led to the suppression of NFL whereas the AhR inhibitor downregulated expression of NFL and reduced phosphorylation of ERK1/2 and p38 (84). However, another study reported that MAPK activation was an alternative mechanism through which TCDD regulated the AhR function, supporting the diversity of TCDD toxicity in a gene- and cell-specific manner (85). Notably, Tan et al. showed that MAPK activation by TCDD could also occur in AhR-negative CV-1 cells as well as in AhR KO mouse embryonic fibroblasts (86). Besides TCDD, another AhR ligand 3-methylcholanthrene was shown to alter the epithelial cell plasticity by a mechanism that involved JNK activation (87). Additionally, in the process of osteoblast formation, indoxyl sulfate (IS) inhibited the ERK and p38 MAPK pathways downstream of AhR. Conversely, resveratrol counteracted the anti-osteogenic effect of IS by inhibiting AhR and associated downstream signaling (88). Moreover, macrophages from chronic kidney disease (CKD) patients exposed to the uremic toxin IS displayed inflammation through activation of the AhR/NF–κB/MAPK cascade, in a process that induced the mature IL-1β production without activating NLRP3 inflammasome (89). Overall, accumulating evidence supports the notion that there is a bi-directional crosstalk between MAPKs and AhR pathways and that AhR ligands can activate one or more MAPKs, depending on the ligand, cell, or tissue types, leading to the nuclear translocation and DNA binding of AhR and target gene transactivation. The interaction between AhR and MAPK pathway is depicted in **Figure 5**.

### Interaction between AhR and epidermal growth factor receptor pathway

EGFR is a crucial receptor tyrosine kinase (RTK), a transmembrane protein belonging to the ErbB family, and it plays a role in embryonic development. Ligand binding leads to the receptor autophosphorylation and formation of hetero- or homodimers and the recruitment of signaling proteins. Downstream signaling pathways involve the MAPK, RAS-RAF-MEK1/2- ERK1/2, AKT-PI3K-mTOR, PKC, STAT, SRC, and NF-κB (90). EGFR is associated with various cancers, often due to mutations that cause continuous activation. The AhR-EGFR interaction was first observed in 1982, suggesting that polycyclic aromatic hydrocarbons (PAHs) not only activated AhR but also inhibited the EGFR binding to its ligand EGF (91). Subsequent reports indicated that exposure to AhR agonists disrupted the binding of radiolabeled EGF to the plasma membrane. The underlying mechanism involved the AhR ligand-mediated enhancement of EGFR internalization and c-Src-mediated phosphorylation or inhibition of the EGFR extracellular domain binding to cognate ligand (92, 93). Unlike the transient effects of PAH, TCDD reduced the EGF-binding capacity of cognate receptors in human keratinocytes rat liver for 4 and 40 days, respectively (94, 95). The study by Vogeley et al. provides in-depth insights into the complex dynamism between AhR ligands and EGFR internalization, by showing that PAHs exposure of human keratinocytes led to a dual-phase increase in EGFR phosphorylation and downstream MEK/ERK signal transduction (92). Notably, dioxin-like compounds like PCB126 and 2,3,7,8-tetrachlorodibenzo-p-dioxin demonstrated similar AhR-dependent and c-Src-driven signaling events, leading to the release of EGFR ligands. These studies establish that dioxin-like compounds bind directly to the extracellular domain of EGFR, thereby hindering receptor tyrosine kinase activation by growth factors (92, 96).

**Figure 6:**
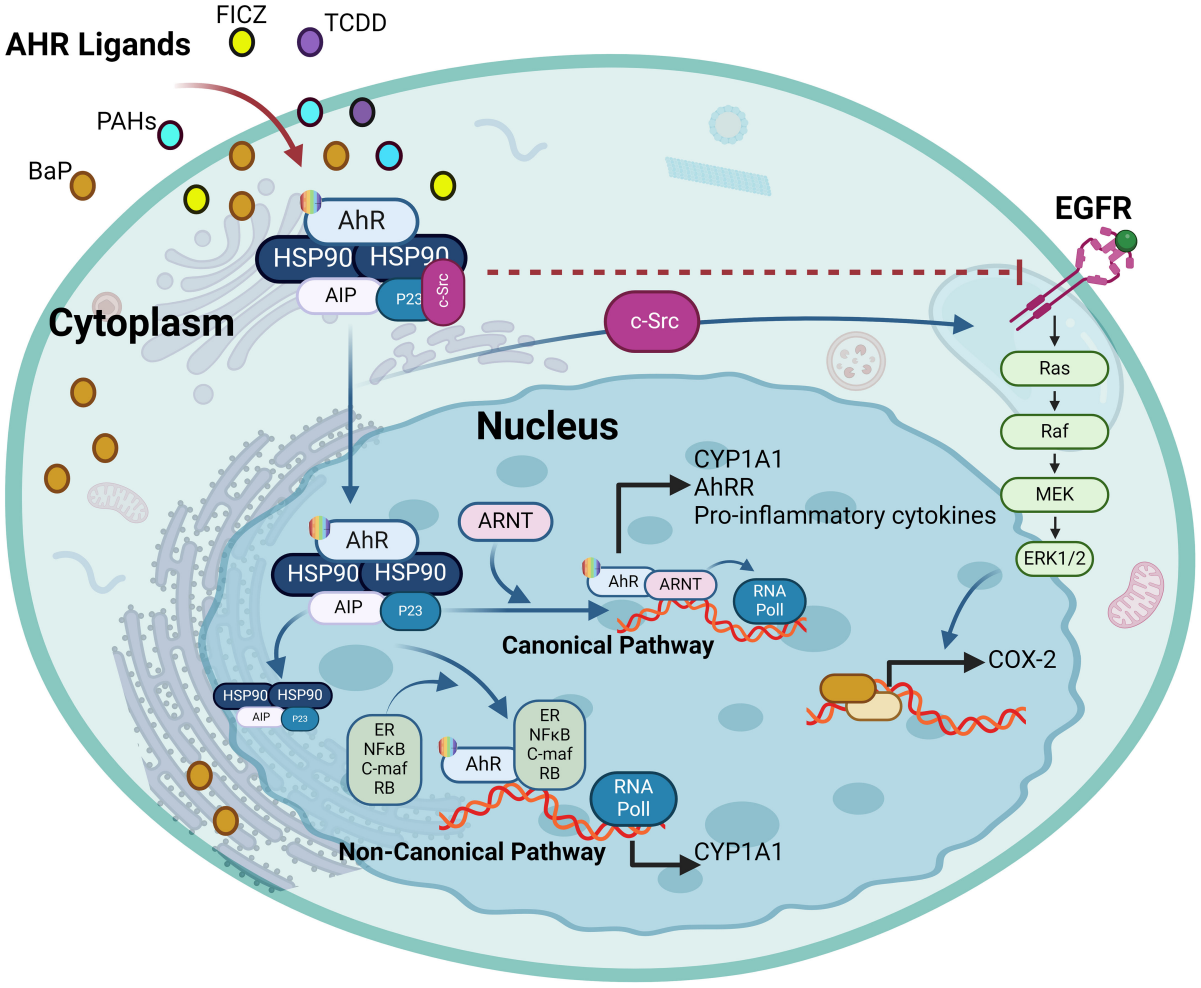
The ligand-activated AhR activates EGFR and downstream signaling. The ligand-driven dissociation of the AhR complex leads to the release of c-Src, which can (I) directly activate the epidermal growth factor receptor (EGFR) by phosphorylating its intracellular domain, and (II) sequentially activate protein kinase C (PKC) and sheddases resulting in ectodomain shedding of cell surface-bound EGFR ligands. In addition, nuclear AhR transactivates genes encoding EGFR ligands, such as amphiregulin (AREG) and epiregulin (EREG) (III). Independently from its mode of activation, i.e. ligand-binding or intracellular phosphorylation, the EGFR monomer changes its conformation from tethered to untethered and forms a hetero- or homodimer leading to activation of downstream signaling pathways like MAPK or JAK/STAT. This illustration was created with Biorender.com.

Notably, the AhR-driven modulation of EGFR function has been investigated in various cancers. In this regard, TCDD-induced EGFR expression changes in breast cancer cells were associated with downstream signaling activation and apoptosis inhibition, whereas this effect was reversed by an AhR antagonist, indicating a tumor-promoting effect of EGFR-mediated AhR signaling (97). The EGFR-AhR interaction has also been linked to cellular response to UVB stress and the development of skin tumors through the mechanism involving UVB-induced formation of the natural AhR ligand 6-formylindolo[3,2-b]carbazole (FICZ) in the skin which leads to c-Src-dependent EGFR internalization and ERK activation (98). This UVB-induced AhR signaling activation and skin tumor formation was associated with the expression of cyclooxygenase-2 (COX-2) and the consequent DNA damage (99). Involvement of the AhR-regulated MMP-1/EGFR signaling in colorectal cancer pathogenesis was indicated as the suppression of MMP-1, a gene induced by AhR, led to the improvement of colorectal cancer by inhibiting EGFR-downstream PI3K/AKT signaling (100). Evidence of the AhR-EGFR pathway involvement was provided by an asthma mouse model study, showing that Benzo(a)pyrene (BaP)-induced AhR activation and ROS elevation led to the increased epithelial TGF-α production and MUC5AC expression, and activation of EGFR/MAPK signaling, thus causing airway obstruction and asthmatic distress (101). Consistent with the role of AhR in lung morbidities, Liu et al. demonstrated that IFN-AhR signaling in COVID-19 patients promoted mucins and the alveolar mucus contributed to hypoxia and inflammation; while inhibiting the AhR signaling resulted in improved lung function in a SARS-CoV-2 mouse model (102). The intricate interplay between AhR and EGFR holds significant implications for therapies directed at the EGFR and associated signaling pathways. Presently, the EGFR targeting monoclonal antibodies or inhibitors are presenting with skin toxicity side effects, necessitating their withdrawal. The dynamic interactions between EGFR and AhR signaling cascades suggest that when formulating EGFR-directed therapies, it may be desirable to also consider AhR as a potential target or co-target. This consideration is vital to reduce the undesired side effects and improve the effectiveness of the treatment. The interaction between AhR and EGFR is illustrated in **Figure 6**.

## Interaction between AhR and JAK/STAT pathway

The JAK/STAT pathway is a critical cascade that transduces signals from the cell surface to the nucleus and is involved in processes such as cell division, cell death, immunity, and tumor formation (103). Dysregulated JAK/STAT signaling is implicated in multiple disease pathogenesis including autoimmunity, chronic inflammatory conditions, and cancer (104, 105). Mechanistically, members of the JAK family bind to intracellular domains of transmembrane cytokines and interferon receptors or receptor tyrosine kinases, such as EGFR. Upon ligand activation, these receptors dimerize or multimerize, initiating JAK-mediated phosphorylation of specific tyrosine residues in the intracellular domains of these receptors. These phosphorylated residues act as docking sites for the Src-homology 2 (SH2) domain of signal transducer and activator of transcription (STAT) proteins. Following JAKs binding to phosphorylated cytokines’ receptors, C-terminal tyrosine residues in STAT proteins are phosphorylated, and this newly-generated SH2-binding motif is then recognized by two STAT proteins, forming a STAT dimer which translocates to the nucleus where it acts as a transcription factor by binding to specific DNA motifs in the enhancer regions of target genes (103). In support of a crosstalk between AhR activation and STAT signaling, the study by Nukaya et al. showed that treating C57BL/6 mice with an AhR agonist 3-methylcholanthrene led to the AhR-dependent suppression of JAK2 expression in the liver, which associated with impaired DNA-binding activity of STAT5 and disruption of the growth hormone signaling pathway (106). Similarly, Takanage et al. reported that the AhR agonist β-naphthoflavone impaired cAMP-induced astrocytic differentiation of C6 glioma cells by inhibiting *IL-6* gene expression and suppressing STAT3 activation (107). These findings corroborate that AhR-mediated signaling modulates the JAK/STAT activity by regulating the expression of cytokines and other pathway components. In this regard, AhR-mediated signaling following ligand binding regulates the expression of various JAK/STAT-stimulating cytokines including IL-2, IL-10, IL-21, IL-22 and others, through different pathways. AhR cooperates with NF-κB subunit RelA/p65 at the IL-6 promoter (108); however, AhR-mediated regulation of cytokines can be context-dependent, resulting in cytokine induction or inhibition, as reported for IL-6 and IL-33 (109–111).

**Figure 7:**
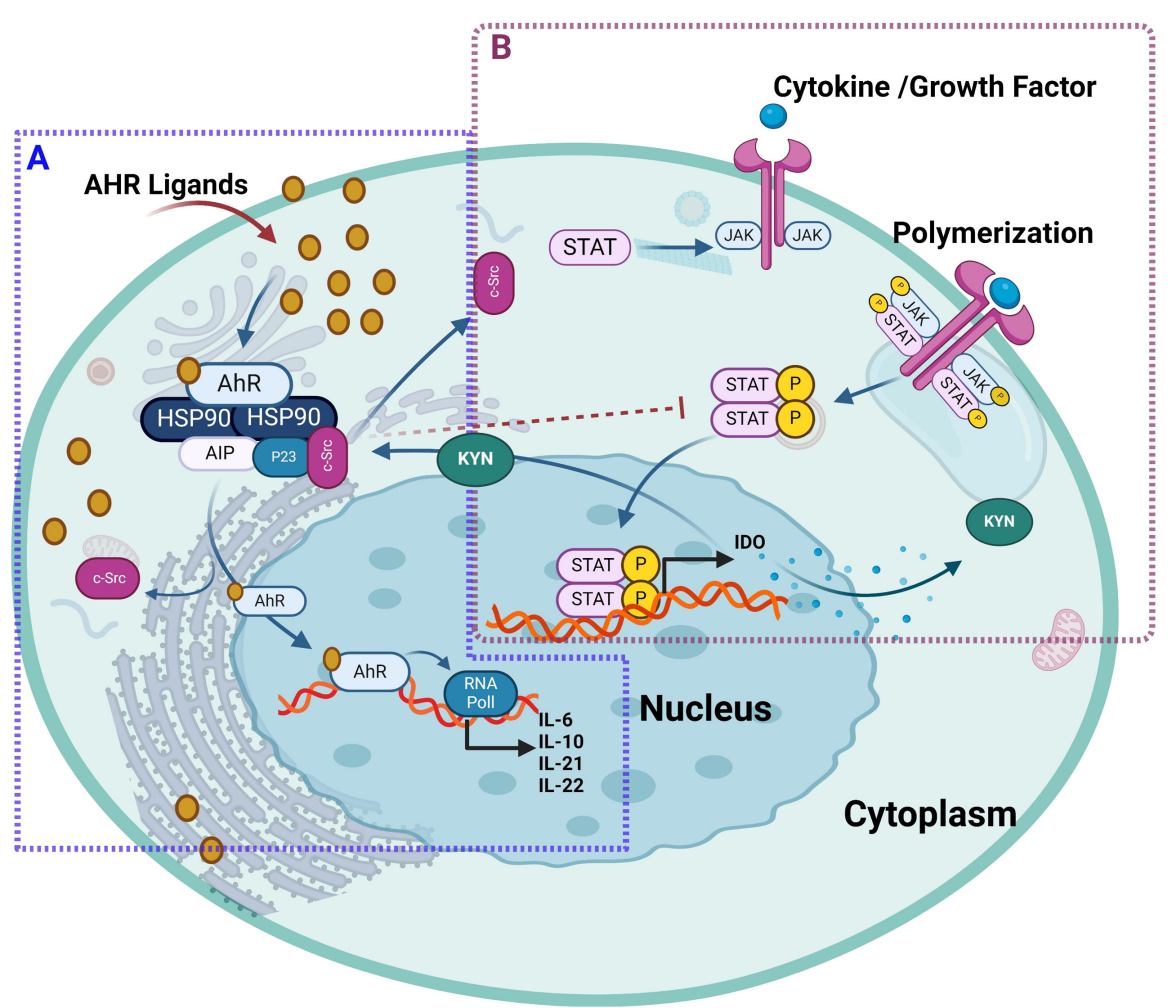
The ligand-activated AhR interacts with JAK/STAT pathway. **(A)** AhR ligand bound to AhR receptor, will translocate AhR into the nucleus and forms a heterodimer to drive transcription of AhR target genes such as CYP enzymes and regulates the expression of different JAK/STAT-stimulating cytokines including IL-2, IL-10, IL-21, IL-22. In addition, both AhR ligand called 3-methylcholanthrene and β-naphthoflavone led to the AhR-dependent suppression of STAT 5 and STAT3 activation expression, respectively. **(B)** Cytokines and growth factors bind to their receptors, leading to receptor dimerization and recruitment of related JAKs. JAK activation leads to phosphorylation of the receptors and formation of docking sites for STAT. Then, STATs dissociate from the receptor to form homodimers or heterodimers. These STAT dimers enter the nucleus, bind to DNA, and regulate transcription to release IDO1 in human chronic lymphocytic leukemia cells. Furthermore, tryptophan is oxidized by IDO1 to N-formylkynurenine, which is further converted by aryl formamidase into KYN. KYN and its metabolites act as AhR agonists that induce immunosuppressive Tregs and simulate TCDD effects. This illustration was created with Biorender.com.

Further in this regard, Rothhammer et al. deciphered the regulatory role of AhR signaling in type I IFN-mediated astrocyte activity and central nervous system inflammation via the mechanism involving IFN-β-associated JAK1 and tyrosine kinase 2 activation and formation of a macromolecular complex including STAT1/2, and IRF-9 (112). In B cells, IL-4 induced AhR expression in a STAT6-dependent manner, albeit the precise molecular mechanism regulating IL4-mediated induction of AhR remains to be fully elucidated (112, 113). It was suggested that at the transcriptional level, STAT proteins played a role in AhR activation by influencing tryptophan metabolism (113). Consistent with this, another study showed that interferon-gamma (IFN-γ) induced IDO1 in human chronic lymphocytic leukemia cells which depended on the JAK/STAT1 signaling. Tryptophan is oxidized by IDO1 to N-formylkynurenine, which is further converted by aryl formamidase into KYN. KYN and its metabolites (kynurenic acid and xanthurenic acid) act as low-affinity AhR agonists. KYN activation of AhR has been associated with the induction of immunosuppressive Tregs, simulating TCDD effects in mice (114, 115). In lung cancer, an autocrine signaling loop mechanism was deciphered, which involved KYN activation of AhR, followed sequentially AhR-mediated IL-6 upregulation, STAT3 stimulation, and KYN-producing IDO1 induction (116). A similar mechanism in the tumor microenvironment induced CD8+ T cell exhaustion via IL-2-mediated STAT5 activation and tryptophan hydroxylase-1 expression, leading to AhR activation. This resulted in the upregulation of inhibitory receptors CD39 and PD-1, and downregulation of IFN-γ and tumor necrosis factor (TNF)-α, causing CD8+ T cell dysfunction (117). The KYN-mediated AhR activation was found to induce immunosuppression and alleviation of symptoms in idiopathic pneumonia syndrome (118). The interaction between AhR and JAK/STAT is illustrated in **Figure 7**.

In summary, dynamic interactions dictate the crosstalk between AhR and STAT family transcription factors at levels of transcriptional regulation, induction of signaling mediators, and protein-protein interactions. Depending on cell types and the STAT signaling mediators involved, the AhR activation may lead to pro- or anti-inflammatory outcomes. Notably, the crosstalk between AhR and JAK/STAT signaling cascades blunts anti-tumor immune responses and promotes malignancies such as lung cancer, melanoma, glioblastoma, and oral squamous cell carcinoma (116, 119, 120). On the contrary, AhR-JAK/STAT crosstalk might have anti-inflammatory consequences, as in inflammatory bowel disease and central nervous system inflammation and neurodegeneration (121). Further research is warranted to unravel the intricacies of AhR-JAK/STAT interplay, providing valuable insights for the development of targeted therapeutic interventions.

## AhR ligands

### Exogenous and endogenous AhR ligands

AhR ligands vary with regard to affinity and reactivity. Currently, most high-affinity AhR ligands are synthetic, including environmental contaminants such as polycyclic aromatic hydrocarbons (PAH) and halogenated aromatic hydrocarbons (HAH) (122, 123). Compared to PAHs, HAHs are metabolically more stable and bind to AhR with increased affinity. Notably, TCDD is a type of HAH that leads to toxicity in the host by activating AhR. In addition, several low-affinity synthetic AhR ligands have also been identified, as described in **Table 1**.

**Table 1:** The common AhR ligands.

Type	Ligand	Ref.
**Endogenous ligands**	Bilirubin	(124)
	Biliverdin	(125)
	Lipoxin A4	(122)
	Cinnabarinic acid(CA)	(126)
	Heme metabolites	(127)
	Indole acrylic acid (IA)	(128)
	Indole-3-acetic acid (IAA)	(129)
	Indole-3-propionic acid (IPA)	(126)
	Indole-3-lactic acid (ILA)	(130)
	Indole-3-aldehyde (IAld)	(131)
	Indole-3-acetaldehyde (IAAld)	(132)
	Indole-3-carboxaldehyde (3-IAld)	(133)
	Indoxyl-3-sulfate (I3S)	(134)
	Tryptamine	(135)
	Skatole (3-Methylindole)	(136, 137)
	Kynurenine (KYN)	(138)
	Kynurenic acid (KA)	(114)
	2-(10H-indole-30-carbonyl)-thiazole- 4 carboxylic acid methyl ester (ITE)	(139)
	6-Formyl indolo (3,2-b) carbazole (FICZ)	(127, 139)
	Xanthurenic acid	(109)
**Synthetic Exogenous ligands**	Benzo[a]pyrene (BaP)	(140, 141)
	Benz(a)anthracene (BA)	(142)
	Polychlorinated biphenyls (PCBs)	(143)
	2,3,7,8-Tetrachlorodibenzo-p-dioxin (TCDD)	
	4-(3-Chloro-phenyl) pyrimidin-2-yl)-(4-trifluoromethylphenyl)-amine (VAF347)	(144)
**Natural Exogenous ligands**	Berberine	(145)
	Carotenoids	(146)
	Flavonoids	(147)
	Indolo[3,2-b] carbazole (ICZ)	(148)
	Indole-3-carbinol	(149)
	Indole-3-acetonitrile	(134)
	Resveratrol	(150)
	2-(indole-3-methane)-3, 3’-diindolylmethane	
	3, 3-diindolylmethane (DIM)	(151)
	Tryptanthrin	(152)
	Curcumin	(153)

Interestingly, a multitude of naturally occurring compounds from dietary sources have been found to directly influence the AhR signaling pathway. Accumulating evidence supports that extracts from vegetables or vegetable-derived materials can stimulate CYP1A1 activity (154, 155). Cruciferous vegetables, including broccoli, cabbage, turnips, cauliflower, kale, and Brussels sprouts, contain the compound glucobrassicin, which is converted into I3C and indole-3-acetonitrile (I3AC) during digestion, which acts as activating AhR ligands (154, 156, 157). Dietary compounds from plant sources including carotenoids, curcumin, and tryptophan, have been found to bind to AhR and trigger target gene expression (123, 153, 158). Flavonoids, present in tea and various fruits, are naturally occurring dietary AhR ligands, with most functioning as AhR antagonists. However, certain flavonoids, such as quercetin, diosmin, tangeritin, tamarixetin, taxifolin, and robinetin are the flavonoids capable of activating AhR (159–161). Importantly, as though these plant-derived AhR ligands are found in micromolar plasma concentrations, they still act as potent AhR signaling modulators (162, 163). Tryptophan metabolism within the gastrointestinal tract involves the following three major pathways (164). In the first pathway, the gut microbiota directly transforms tryptophan to yield various AhR-activating ligands, such as indole, tryptamine, indole acrylic acid, indole-3-acetamide, indole-3-aldehyde, indole-3-acetic acid, indole-3-propionic acid, indole-3-lactic acid (ILA), and indole-3-pyruvate. Indole further metabolizes into biologically-active compounds like dioxindole, indoxyl-3-sulfate, and indole-3-propionic acid (165). Thus, the intestinal commensal microflora is a continual source of potential AhR-activating ligands, underscoring the role of AhR as a sensor of the gut microbiota communities and as a regulator of host-microbe homeostasis (134). In the second pathway called the KYN pathway (KP), commonly operative in immune and epithelial cells, IDO1 activity generates KYN (109, 166), which is a precursor for potent AhR ligands including kynurenic acid, xanthurenic acid, and cinnabarinic acid, with effects on neurotransmission, inflammation, and immune responses (166). In the third pathway, called the serotonin 5-hydroxytryptamine (5-HT) production pathway, tryptophan hydroxylase 1 generates the AhR activating ligands (167). Butyrate supplements improved arthritis in mice by altering microbiota and favoring the production of 5-hydroxyindole-3-acetic acid (5-HIAA), a serotonin-derived metabolite that reduces the severity of arthritis (168). In addition to biliverdin, unconjugated bilirubin (UCB) also acted as an AhR agonist and induced target gene expression (169, 170), while bilirubin activated AhR in Th17 cells and stimulated the production of CD39 and exerted an immunosuppressive effect to alleviate inflammation in an experimental model of colitis in mice (43).

## Role of AhR in immune regulation

Immune regulation is critical in ensuring that the immune system responds appropriately to threats without causing excessive inflammation or autoimmunity. The following key immune effector cells modulate the activity of the immune response, maintain homeostasis and prevent the pathological conditions: (A) Macrophages are crucial in various physiological functions, including development, tissue repair, immunity and maintaining internal environment stability (171). (B) Certain subsets of DCs also contribute to immune regulation. Regulatory dendritic cells (regDCs) can induce Tregs and produce anti-inflammatory cytokines (172, 173). (C) Another key player in immune regulation is the myeloid-derived suppressor cells (MDSCs). These cells are a heterogeneous population of immune cells that expand during cancer, infection, and inflammation, where they suppress T- cell activation and promote tumor progression or chronic infection persistence (174). (D) NK cells, known for their cytotoxic capabilities, are the innate immune lymphoid cells that orchestrate host defense against pathogens and a range of cancers (175). (E) Innate lymphoid cells (ILCs) are the innate counterparts of CD4+ Th lymphocytes, regulating immune responses and maintaining immune homeostasis, particularly in the mucosal linings of the intestine and lung. (F) Tregs play a role in suppressing immune responses, thereby preventing autoimmune diseases and maintaining tolerance to self-antigens. (G) B-lymphocytes play a key role in the adaptive immune response through the secretion of immunoglobulins (176).

### AhR-mediated regulation in macrophages

Macrophages play pivotal roles in a wide array of physiological functions including development, tissue repair, immunity, and maintenance of internal environment stability (171). After injury, macrophages undergo activation and colonization at the site of injury, and orchestrate diverse functions including inflammation, wound healing, fibrosis, decomposition and regeneration, as well as anti-inflammatory and anti-fibrotic activities (177). Based on the production of nitric oxide synthase and arginase, macrophages are classified into pro-inflammatory M1 and anti-inflammatory M2 phenotypes (178). AhR, in concert with macrophage-dependent cellular pathways, regulates macrophage polarization into pro-inflammatory or anti-inflammatory phenotypes (179). Additionally, monocytes’ differentiation into macrophages and the interactions between macrophages and other immune cells are also modulated by AhR signaling (140).

Substantial evidence supports existence of a functional 5-HT/5-HT_2B_/AhR axis in human macrophages (17, 180). Serotonin (5-HT) promotes AhR activity and drives anti-inflammatory targets expression in a 5-HT_2B_-dependent manner (180). Agonist ligands such as PCB126 and FICZ promote proinflammatory M1 macrophage polarization (140). However, another study showed that FICZ induced AhR activation and transcriptional regulation of miR-142a, leading to HIF-1α inhibition, thus suppressing M1 macrophage polarization (181). These findings underscore the role of AhR in immune regulation, as a transcription factor and also through non-genomic signaling pathways. Together, these mechanisms regulate macrophage polarization into anti-inflammatory or pro-inflammatory phenotypes, with implications for disease outcomes.

### AhR-mediated regulation in dendritic cells

DCs are professional antigen presenting cells and play pivotal roles in the innate and adaptive immune responses. DCs constitutively express AhR, and its activation leads to their differentiation from monocytic precursors, through the mechanism favoring Blimp1 expression (172, 173). AhR regulation diminishes pro-inflammatory T cell polarization and promotes differentiation of anti-inflammatory regulatory Tregs. It was shown that AhR agonists stimulated the expression of IDO1 and IDO2 in DCs, leading to KYN production and Foxp3+ Treg differentiation (182). Likewise, another study demonstrated that indole-3-propionic acid (IPA)-mediated AhR activation led to reduced IFN-γ production by DCs and promoted IL-10 producing T-cell differentiation in inflammatory bowel disease (IBD) in mice. Increased numbers of CD103^+^CD11b^−^ anti-inflammatory DCs found in the mesenteric lymph nodes explained the reduced severity of colitis in the mice (132). Overall, these studies highlight the anti-inflammatory effects of AhR in DCs.

AhR signaling was also shown to influence the function of DCs, by promoting their activation and antigen presentation, and the mechanism was AhR-dependent as such effects were absent in AhR-deficient DCs (183). At a functional level, the AhR loss in DCs resulted in a more aggressive experimentally-induced colitis, showing that AhR signaling played a role in the regulation of a functional intestinal epithelial barrier and mucosal immunity (184).

It is also notable that AhR-mediated regulatory signaling was shown to have paradoxical effects. Quintana et al. showed that AhR activation by its endogenous ligand 2- (1′H-in-dole-3′-carbonyl)-thiazole-4-carboxylic acid methyl ester (ITE) led to decreased expression of major histocompatibility complex (MHC) class II and co-stimulatory molecules on splenic DCs and T cells, thus promoting the induction of tolerogenic DCs and FoxP3^+^ Tregs that suppressed experimental autoimmune encephalomyelitis (185). While, another study indicated that AhR-deficient (AhR^−/−^) DCs expressed increased levels of MHC class II and co-stimulatory CD86 molecules, and hence the AhR loss was associated with increased Th2 cell activation and severe pro-inflammatory allergic responses (186). A negatively regulatory role of AhR in DC immunogenesis was suggested as in the presence of CpG or LPS, AhR^−/−^ DCs induced reduced KYN and IL-10 expression (187). It is reasonably speculated that different AhR ligand types might differentially impact the DCs phenotypes and function. Interestingly, a recent study showed that three different AhR ligands including FICZ, indoxyl 3-sulfate (I3S), and BaP had different modulatory effects on DC biology and unlike FICZ or I3S, the BaP induced a tolerogenic response in LPS-primed DCs (188). Taken together, these findings suggest an immunoregulatory role of AhR activation in DCs and the impact on their functional responses.

### AhR-mediated regulation in myeloid-derived suppressor cells

MDSCs, generated during pathological conditions (189), exert immunosuppressive functions. Recent investigations have revealed that TCDD-induced activation of AhR within the peritoneal cavity triggers the mobilization of MDSCs with immunosuppressive capabilities (174). Treatment of mice with an AhR antagonist (CH223191) or CXCR2 receptor antagonist (Sch527123) significantly reduced the TCDD-induced MDSCs, underscoring the dependence of TCDD-mediated immunosuppression on AhR signaling in MDSCs (174). Furthermore, TCDD-induced AhR-mediated mobilization of MDSCs relied on the CXCR2 expression as the blockade of CXCR2 diminished the TCDD-mediated MDSCs induction (190). A recent study showed that the defective AhR activation led to impaired regulation of polymorphonuclear (PMN) MDSCs in a mouse model of experimental Sjögren’s syndrome (ESS) (191). Dietary supplementation with indole-3-propionic acid (IPA), a tryptophan metabolite that activates AhR, promoted the PMN MDSCs differentiation and CD4+ T-cell inhibition, indicating that IPA-induced AhR activation restored PMN MDSCs function in ESS (191) The AhR-associated mechanisms that drive MDSCs regulation and suppressor functions in Sjögren’s syndrome have been discussed in a recent review (192).

### AhR-mediated regulation in NK cells

NK cells are cytotoxic innate immune lymphoid cells that orchestrate host defense against pathogens and several types of cancers. By engaging in reciprocal interactions with macrophages, T-lymphocytes, DCs, and endothelial cells, NK cells act as key players in immune regulation (175). Emerging evidence now supports that NK cells can also manifest adaptive modulations such as antigen-driven clonal expansion and induction of long-lived memory and thus play regulatory roles in both innate and adaptive immunity (193). Notably, AhR signaling is critical to shaping innate and adaptive immunity and plays a key role in the development of NK cell precursors. The underlying molecular mechanisms by which AhR affects NK cell differentiation and function remain largely elusive. The circulatory mature NK cells typically lack in AhR expression, and this inhibition is induced by STAT3 signaling during IL-21-driven NK cell activation and proliferation, in parallel with the upregulation of CD56 expression. In NK cells, AhR regulates genes that are associated with a wide variety of signaling and metabolic pathways, including the oxidative stress responses (194).

Notably, the peripheral human NK cells show differential susceptibility to AhR modulation. In this regard, CD56^bright^ NK cells were found to highly express AhR mRNA and had increased sensitivity to AhR ligands (195). Conversely, AhR mRNA expression gradually declines as NK cells exhibit a more mature phenotype, characterized by the CD56^dim^ phenotype. Additionally, AhR ligands play a role in modulating CD56^bright^ NK cell surface receptors and cytokine secretion (195). AhR emerges as a critical regulator of NK cell migration, and NK cells deficient in AhR expression exhibit diminished capacity for *in vivo* migration. Importantly, Shin et al. showed that AhR bound with the *ASB2* gene promoter and the interaction with the agonist FICZ induced ASB2-dependent degradation of filamin A in NK cells which led to promoted migration of primary NK cells (196).

### AhR-mediated regulation in the ILCs

ILCs are the innate counterpart of CD4+ Th-lymphocytes that regulate immune responses and maintain immune homeostasis, especially in the mucosal linings of the intestine and lung (197). Based on transcriptional circuitry and effector functions, ILCs are classified into three main groups (198), including (i) Group 1 ILCs (ILC1 cells) which resemble Th1 cells (199); (ii) Group 2 ILCs (ILC2 cells) which are characterized by the expression of GATA3 transcription factor – a master regulator of Th2 cells involved in production of IL-5 and IL-13 by this Th-lymphocyte subset (200); and (iii) Group 3 ILCs (ILC3 cells) which mimic Th17 cells and express RORγt transcription factor and produce cytokines including IL-17 (201).

AhR signaling plays a critical role in regulating ILC function and AhR is highly expression in the gut ILC2 subset (202). AhR signaling inhibits the expression of IL-33R, IL-5, IL-13, and the transcription factor GFI1. While activation of AhR suppresses the function of ILC2 subset, it ensures the maintenance of ILC3 subset. Thus, the AhR pathway maintains the balance between intestinal ILC2 and ILC3 effector populations, contributing to a protective immune response against local pathogens (202). It is noteworthy that the AhR pathway controlled the phenotypic changes in ILCs populations found in the inflamed terminal ileum of individuals with celiac disease, as indicated by the ILC3 to ILC1 shift and a downregulation of AhR expression in the intestinal ILCs (203).

The role of AhR in the intestinal ILC3s has been thoroughly studied, with the following key findings reported. Firstly, AhR is critical to the survival of ILC3s (204). The underlying mechanisms that promoted the survival and maintenance of ILC3s included the expression of IL-7/IL-7R pathway and anti-apoptotic genes (205). Interestingly, AhR-deficient ILC3s exhibit reduced Ki67 expression and diminished proliferation (206). Secondly, AhR acts as a potential regulator of ILC3s development (206). Mechanistically, AhR induction by Runx3 and its downstream target RORγt led to promote the development of ILC3s (207), while the C2H2 zinc finger transcription factor Ikaros negatively regulated gut ILC3s and inhibited their expansion by suppressing AhR expression (208). Other mechanisms of AhR-mediated regulation of ILCs involved the increased transcription of Notch 1/2 and stabilization of c-Kit expression (209, 210); however, AhR also regulated ILCs through the Notch-independent mechanisms (210). Lastly, AhR signaling also modulated IL-22 production by ILC3s (211). In AhR-deficient mice, ILC3 levels were significantly reduced, leading to cryptic cap dysplasia, isolated lymphatic follicles, and inadequate intestinal IL-22 production (209). It was suggested that tryptophan metabolites derived from the gut microbiome acted as activating AhR ligands and promoted intestinal homeostasis via increased IL-22 production by ILC3s (212). Additionally, microbiome-derived short-chain fatty acids (SCFAs) can also upregulate AhR expression and enhance IL-22 production. Notably, Yang et al. showed that a dietary supplementation with SCFAs boosted the IL-22 production and protected mice from intestinal inflammation, following infection by *Citrobacter rodentium* (213). Moreover, individuals with alcoholic hepatitis exhibited low fecal levels of intestinal microbiota-derived tryptophan catabolite and an AhR ligand, indole-3-acetic acid (IAA) (214, 215). Supplementation with IAA activated AhR signaling, increasing ILC3 populations and IL-22 production in the gut, thereby shielding mice from alcohol-induced steatohepatitis (216).

### AhR-mediated regulation in lymphocytes

#### Immunoregulation in T-lymphocytes

Recent investigations support the pivotal role of AhR as a master regulator of the adaptive immune responses, particularly in Tregs and Th17 cell differentiation in response to a diverse array of endogenous, dietary, microbial, and environmental ligands (217). Tregs, characterized by the expression of Foxp3 transcription factor, are vital in preserving immunological balance and maintaining peripheral self-tolerance (218). Common AhR ligands such as TCDD, KYN, PB502, ITE, and FIZC have been identified to induce Foxp3+ Tregs expansion by activating AhR-mediated signaling (17, 217, 219, 220). Interestingly, it was shown that TCDD-mediated AhR activation in the presence of TGFβ1 induced SMAD1 and stabilized Foxp3 expression in human Tregs (53). AhR activation by its ligand has been reported to regulate the Treg and Th17 cell development (37). In murine Th17 cells, AhR signaling countered STAT1 activation and positively regulated Th17 cell differentiation and development (221). The tissue specific effects and differential contribution of AhR agonists associated with commensal flora can modulate AhR-mediated immune regulation in Tregs. Additionally, other AhR ligands such as Baicalein, NOR, Alpine, and Laquimod metabolites were also found to directly influence Treg differentiation of Tregs, offering potential alleviation of colitis through AhR pathway activation (36, 37, 118, 222, 223). Of note, AhR activation was shown to directly modulate Foxp3 transcriptional regulation by binding to its promoter or indirectly by regulating downstream signaling through TGF-β (37). Xiong et al. demonstrated that AhR signaling directly upregulated Foxp3 transcription and promoted the Tregs activity by influencing CD4+ T-cell intestinal homing through G protein-coupled receptor (GPR)-15, under steady-state and inflammatory conditions (224). Besides influencing the intestinal homing, AhR signaling also modulated function of Tregs by suppressing pro-inflammatory cytokines’ production by Tregs *in vivo* (225). It is plausible that different sub-populations of Tregs could have differential levels of AhR expression and hence show the variable sensitivity to immune regulation by AhR ligands. In line with this argument, Foxp3+ Tregs expressing the co-inhibitory molecule TIGIT were found to be more susceptible to modulation by AhR agonists than other Treg subsets (226). Overall, these findings suggest an AhR-driven regulation in functional Tregs, as shown by protective effects of AhR activation in morbid conditions including colitis (227), diabetes (228), and experimental autoimmune encephalomyelitis (185).

In addition to Foxp3+ Tregs, another well-characterized regulatory T-lymphocyte subset is known as type-1 regulatory T (Tr1) cells which are Foxp3− CD4+ T lymphocytes that produce IL-10 and play non-redundant roles in controlling inflammation (49, 229). Tr1 cells also produce IL-21 which has an autocrine effect to support Tr1 cell stabilization and differentiation (230, 231). IL-27 also promotes Tr1 cell differentiation (232, 233). Importantly, AhR was identified as a metabolic regulator of Tr1 cell differentiation (34), and it synergized with cMaf to regulate the production of IL-10 and IL-21 in Tr1 cells (52, 53).

Additionally, the AhR-STAT3 cooperativity leads to increased expression of CD39 which controls the suppressive activity of Tr1 cells (234). Similarly, the AhR-HIF1α interaction regulated glucose metabolism in Tr1 cells (34), and it is speculated that such interactions could also contribute to the key role of these transcription factors in the differentiation of other T-cell subsets, such as Th17 cells (235). Th17 cells are marked by the expression of IL-17A cytokine and RORγt transcription factor (236). IL-1β, IL-6, and IL-23 induce the expression of both IL-17A and RORγt in Th17 cells (237). AhR exhibits high expression in CD4+ RORγt+ Foxp3− Th17 cells; however, the precise mechanisms that drive the activation of AhR and resulting ‘regulation of Th17 differentiation and function remain unclear. Several studies have shown that Th17 cell differentiation is initiated by combined stimulation with TGF-β and IL-6 or with TGF-β and IL-21 (238, 239); while TGF-β promotes the expression of IL-22 by Th17 cells (240, 241). A recent study by Minns et al. unveiled that a broad immune modulator, vinblastine, triggered the Smad2/3 and STAT3 phosphorylation which depended on AhR-mediated signaling. Combined with TGF-β1, vinblastine suppressed IL-2/T-bet expression, favoring a Th17 over Th1 phenotype (242).

The dietary indole derivative I3C was found to elevate the CD4+ RORγt+ Foxp3− Th17 cell numbers in the intraepithelial layer, lamina propria, and Peyer’s patches of the small intestine, improving the progression of type 1 diabetes (T1D) in non-obese diabetic (NOD) mice (243).

In addition to CD4+ T-cells, AhR signaling also modulated CD8+ T-cell responses. To this effect, TCDD suppressed CD8+ T cell differentiation and proliferation in influenza infection, While the precise molecular mechanisms largely remained elusive (244). Liu et al. reported that sustained IL-2 elevation in tumor microenvironment led to STAT5 activation in CD8+ T- cells, and the resulting increased levels of tryptophan metabolite 5-hydroxytryptophan (5-HTP) caused activation and nuclear translocation of AhR. These IL2-driven immunomodulatory changes upregulated CD8+ T-cells inhibitory receptors and caused T-cell exhaustion in murine and human tumors (117). Interestingly, several studies corroborated that the transport of AhR agonist KYN to CD8+ T-cells by a common neutral amino acid transporter called solute carrier family 7 member 8 (SLC7A8) and proton-dependent amino acid transporter 4 (PAT4) activated AhR and upregulated the expression of programmed cell death-1 (PD-1), thereby causing CD8+ T cell exhaustion and dysfunction (245, 246).

AhR activation also plays a critical role in immunotoxicity including the age-related thymic involution by regulating development and differentiation of T cells within the thymus and influencing the balance between thymic T-cells and Tregs (1, 247, 248). In addition to the T cell-Treg imbalance, increased AhR activation with advancing age also suppresses the thymic epithelial cells (TECs) that support the T-cell development, leading to reduced T-cell maturation and selection (1). AhR activation leads to the increased apoptosis of thymocytes and TECs. In addition to aging, chronic exposures to environmental pollutants such as BaP/PAHs (249) and TCDD/dioxins (250–253) can also promote the AhR-mediated thymic involution through diverse mechanisms involving premature emigration of T-cell progenitor, thymocyte proliferation arrest, and thymocyte loss. It was also reported that CD11c^+^ DCs played a crucial role in TCDD-induced thymic involution and disruption of T-cell development and differentiation within the thymus (254). On the contrary, AhR activation in murine TECs was found to support the thymus regeneration whereby AhR signaling enhanced the expression of IL-22RA1 at transcriptional level, resulting in thymus regeneration and improvement of chronic GvHD in the mouse model (255).

As opposed to its deleterious effects, the AhR activation in CD8+ T cells was also found to have host-beneficial effects. Genome-wide analyses revealed that AhR signaling suppressed the circulatory but promoted the resident memory core gene programs (256). Notably, AhR is highly expressed in human intestinal intraepithelial CD8+ T lymphocytes (IELs), and thus the AhR activation leads to increased differentiation and granzyme B production, boosting the IELs-mediated immunity (256). Similarly, AhR is also highly expressed in tissue-resident memory CD8+ T cells (TRMs) and the skin TRMs play a dynamic role in host defense against microbial pathogens (257, 258). Indeed, AhR activation by its agonists ILA or TCDD was shown to induce CD4+ CD8*α α*+ double positive IELs which fortified tolerance to the dietary antigens (259).

Like in αβ T cells, AhR is also expressed in all innate immune γδ T cells (260), which are enriched in most peripheral tissues including the lungs, intestines, and skin, and regulate the first line of immune defense and homeostasis at epithelial surfaces (261). Numerous studies show that AhR activity is critical to the functioning of γδ T cells and the AhR deficiency led to a dramatic reduction in the intestinal Vγ5 and the cutaneous Vγ3 γδ T cells (260), as well as suppressed the production of IL-17 and IL-22 (262–264).

Immunoregulation in B lymphocytes

B lymphocytes play a key role in the adaptive immune response through the secretion of immunoglobulins (176). While the precise mechanisms governing the regulation of B-cell responses by the environmental sensor AhR remain incompletely understood, previous studies have shed light on its immunoregulatory role in B-cells (265). Of note, Vaidyanathan et al. showed that following B-cell receptor cross-linking, AhR was highly induced in B cells and played a critical regulatory role in activation-induced cell fate outcomes, such as plasma cell differentiation and negatively regulating class switch recombination by decreasing expression of PR domain zinc finger protein 1 (*Prdm1*), B-lymphocyte induced maturation protein 1 (*Blimp1*), and activation-induced cytidine deaminase (*Aicda*), respectively (266). It indicated that AhR acted as a molecular rheostat for cell fate decisions in B lymphocytes, controlling effector responses to facilitate the optimal recall responses (266). AhR activation positively regulated gut migration markers on mouse B cells (267). Activation of AhR also negatively regulated differentiation and maturation of B cells in the murine model (268). Ligand-induced AhR activation repressed B-cell differentiation and transcription of lineage-related genes which impeded the transition of mature B cells into antibody-producing plasma cells (266). Specifically, TCDD-mediated AhR activation inhibited the generation of early B-cells and pre-B-cells, through the AhR-mediated transcriptional regulation of early B-cell factor 1 (EBF1) and paired box gene 5 (PAX5) (269). Mature B cells are highly sensitive targets for TCDD-mediated AhR activation, resulting in their compromised ability to secrete immunoglobulin M (IgM) and immunoglobulin G (IgG) (270). An interesting study by Rosser et al. found that dietary supplementation with SCFA butyrate suppressed arthritis in mice through elevated production of 5-hydroxyindole-3-acetic acid (5-HIAA), an endogenous AhR agonist, which activated the AhR-dependent gene transcription and regulatory B cells (Breg) function to ameliorate experimental arthritis (168).

**Figure 8:**
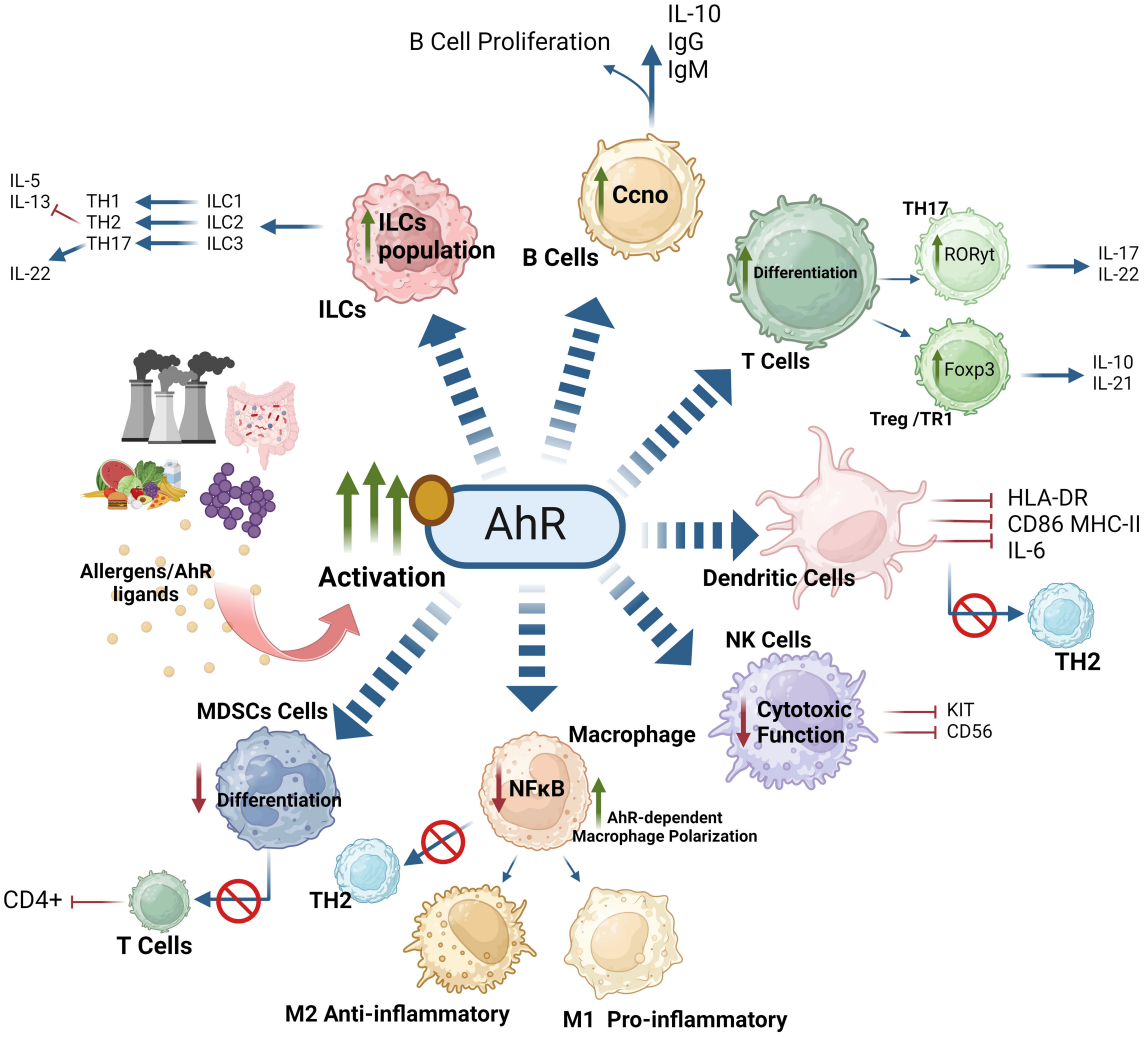
AhR-activation orchestrates both positive and negative regulatory effects on immune cells. AhR-mediated response to exogenous or endogenous ligands influences the activity of adaptive and innate immune responses. AhR signaling affects the polarization of macrophages, suppressing the function of NK cells. Moreover, AhR signaling inhibits the expression of IL-5, IL-13 and enhances the production of IL-22 by ILC3. AhR signaling also effects T cell and B cell differentiation. AhR activation impairs regulation of myeloid derived suppressor cells (MDSCs) and influences the activation of dendritic cell functions. These effects of AhR ultimately imbalance the M1/M2 polarization and Th17/Treg balance. This illustration was created with Biorender.com.

In essence, AhR activation orchestrates both positive and negative regulatory effects in various immune cell types. In macrophages, AhR activation and nuclear translocation leads to both pro- and anti-inflammatory responses by modulating macrophage-dependent pathways and influencing the M1/M2 macrophage polarization from naïve precursors. AhR activation drives monocyte differentiation into DCs, leading to DC activation, co-stimulation, and proficient antigen presentation in response to various AhR ligands. In MDSCs, AhR activation leads to CXCR2-dependent activation and immune suppression. Regarding CD4+ T cells, AhR activation promotes the differentiation of Tregs and Th17 cells by directly increasing Foxp3 transcription and homing in Tregs as well as expression of IL-17A and IL-22 in Th17 cells. In CD8+ T cells, AhR plays a dual role, acting as both a promoter of T-cell exhaustion and as a critical factor in the maintenance of TRMs. Regarding ILCs, AhR activating signaling drives the phenotypic modulation to ensure the survival of ILC3s and promote the IL-22, contributing to immune homeostasis and response modulation. In B cells, AhR activation counteracts mouse B-cell differentiation and development, suppressing the generation of early B-cells and pro-B cells as well as preventing the development of plasma cells from mature B cells. However, in Bregs, AhR activation enhances immunosuppressive functions. The AhR-dependent regulation in various immune effector cell populations is illustrated in **Figure 8**.

## AhR as a therapeutic target

The discovery of AhR ligands with well-documented health benefits and potential pharmaceutical properties has spurred research into developing new therapeutic strategies, targeting AhR-mediated modulation for various diseases, including specific tumors, immune disorders, autoimmune, and inflammatory conditions. Additionally, AhR modulation is being explored to enhance hematopoietic stem cell production. The development of AhR-targeting drugs includes selective AhR modulators (SAhRMs) or ligands that exhibit tissue-specific AhR agonistic or antagonistic activity (271). Tissue-specific variations in agonistic or antagonistic activity stem from ligand-induced conformational changes in the receptor, along with subsequent interactions with essential co-activators and co-repressors involved in target genes’ regulation.

Given the ‘widespread AhR expression in tumors, it emerges as a promising drug target that can be effectively regulated in a cell type-specific manner. ‘Molecules with antagonistic AhR activity are being investigated as potential candidates for treatment of various cancers. Notable antagonists including alpha-naphthoflavone (272) and the high-affinity AhR antagonist StemRegenin 1 (SR1) have shown promise in enhancing the proliferation of human hematopoietic stem cells *in vitro* (273).

Other AhR ligands, like bilirubin and ITE, have demonstrated potent effects on the immune system with potential therapeutic implications. Bilirubin treatment in mice has been found to suppress the development of autoimmune diseases, highlighting its immunomodulatory properties (274). ITE, a synthetic potent agonistic ligand of AhR, has shown efficacy in reducing OCT4 levels, inducing the differentiation of stem-like cancer cells, and attenuating their tumorigenic potential in xenograft tumor models (275). In ovarian cancer, ITE has been observed to regulate cancer cell proliferation and migration via AhR, suggesting its potential for optimizing ovarian cancer therapy (276).

Several human dietary components including flavonoids, quercetin, kaempferol/campherol, and apigenin have been shown to have either agonistic or antagonistic effects on AhR in different tissues and thus the AhR provides a vital link between dietary components and maintenance of intestinal immunity (277). Ito et al. demonstrated that certain tryptophan derivatives, such as I3C, specific to cruciferous vegetables were converted into high-affinity AhR ligands and triggered the AhR signaling (156). Notably, Li et al. showed that a dietary switch from a vegetable origin to a synthetic feed in mice led to a dramatic reduction in IEL numbers with increased Bacteroides colonization and a higher susceptibility to epithelial damage and the detrimental changes were rescued by replenishing I3C in the diet (260). Pre-clinical studies using I3C as a chemo-preventive agent have yielded promising outcomes (278, 279). I3C has also been successfully investigated in phase I clinical trial for its tolerability and efficacy as a dietary supplement in women (280).

Human gut and cutaneous microbiota generate metabolites such as indirubin and indole-3 aldehyde which promote AhR signaling, with improvement in barrier functions (281, 282). A cutaneous microbiota-derived metabolite quinolinic acid was found to negatively regulate activation of the AhR-NLRP3 inflammasome signaling in psoriasis (283). Furthermore, AhR-based manipulations offer reprogramming advantages in various immune effector populations. Notably, AhR stimulatory signaling regulates the cellular differentiation and immunosuppressive functions of polymorphonuclear MDSCs and Bregs, impacts the differentiation of Tregs and Th17 cells, and it also contributes to the maintenance of TRMs and survival of ILCs, especially the IL-22-producing ILC3s. AhR activation in the gut has led to the improvement of permeability, gut immunity, and inflammation (284). AhR modulation by 3,3'-diindolylmethane generated neuroprotective responses against LPS-induced inflammation and neuronal hypoxia using *in vitro* and *in vivo* models of Parkinson’s disease (285). The KYN-AhR pathway emerges as a key player in brain neuronal damage and represents a therapeutic target in stroke (286). These diverse roles highlight AhR’s dynamic impact on immune regulation and underscore its great potential as a therapeutic target for neuro-immune disorders.

## Conclusions and perspectives

While initially recognized for its involvement in cellular responses to foreign substances, AhR has emerged as a pivotal player in human physiology, impacting development, organ function, and immune metabolic equilibrium. AhR deficiency or dysregulated activity has been implicated in compromised intestinal permeability and integrity. Emerging evidence shows that AhR plays significant roles in cytokine expression and the differentiation and functioning of immune effector cells across a broad spectrum, with an impact on both innate and adaptive immune responses. Several lines of research suggest that the AhR pathway plays a key modulatory role in the immune response. Notably, M1/M2 macrophage polarization, maintaining the Th1/Th2 balance and CD4+ CD25+ Foxp3+ Tregs, ILCs, NK cells, and DCs are among the immune effector cells that are mainly impacted by the AhR activation by dioxins and other ligands. Since the AhR interacts with a variety of exogenous ligands, it serves as a potential target for small molecule-based therapies. Nonetheless, most AhR agonists and antagonists are not tissue-specific and require further development to gain optimal effects of such therapeutic interventions. **Table 2** summarizes the role of AhR activation in immune diseases and pathways implicated. Specifically, the clinical applications of AhR-related therapies need more attention. It is notable that the ubiquitous expression of AhR in a wide variety of tissues and cell types can be challenging in targeting AhR signaling pathway for a desirable therapeutic outcome. Given that, cell-specific ligand delivery might be required to target AhR pathway. In this regard, nanoparticles might be used for delivering AhR modulators to targets in specific tissues and cell types (314). More importantly, it is noteworthy that many functional investigations have essentially relied on using rodent models, and the physio-metabolic differences between species may constrain the direct application of the findings to human perspectives. Thus, further research is warranted to uncover additional roles of the AhR, more relevant to human pathophysiology. Indeed, AhR emerges as a key regulator of the immune responses, and it also represents a promising target for novel immunotherapeutic modulations.

**Table 2:** The role of AhR activation in immune diseases and pathways.

**Disease**	**AhR ligands**	**Role pathways**	**Ref.**
**LPS-induced septic shock**	3-MC, TCDD, FICZ, KYN	IDO/TDO activation; TRP metabolism	(287–289)
**Listeria Monocytogenes**	TCCD, FICZ	ROS formation and cytokine expression	(290)
**Herpes-simplex virus-induced ocular Infection**	TCDD	Unclear but decreased numbers of inflammatory IFN-γ+ secreting CD4+ T cells (Th1) and Th17 cells	(55)
**Multiple Sclerosis**	TCDD, I3C, DIM, FICZ	FOXP3 expression, Treg expansion, Th17 expansion	(112, 234, 291–294)
**Inflammatory Bowel Disease**	TCDD, NOR, FICZ	Pro-inflammatory cytokine expression, Th17 differentiation, Treg differentiation, NLRP3 inflammasome expression	(63, 293, 295–297)
**Rheumatoid Arthritis**	TCDD, FICZ	Pro-inflammatory cytokine expression, NF-κb	(298–300)
**Psoriasis**	FICZ, tapinarof	Pro-inflammatory cytokine expression, keratinocyte interaction with adaptive immune system	(301–304)
**Atherosclerosis**	TCDD, BaP	Pro-inflammatory cytokine expression, reactive oxygen species, TCF21 interactions	(305–309)
**Type 1 diabetes mellitus**	TCDD	Th17 expansion, Foxp3 expression and Tregs (type 1 regulatory T cells)	(310)
**Systemic Lupus Erythematosus**	KYN, FIZC, PAHs, dioxins, and TCDD	T cell differentiation and activation, suppress DNA methyltransferase 1 activity in CD4+ T cells of SLE patients and induce CD4+ T cells methylation-sensitive gene hypomethylation, affect the differentiation of Th17 and Treg *in vitro*	(311)
**Asthma**	TCDD, BaP, I3S, Kynurenine, FICZ, lipoxin	Mitochondrial dysfunction and reactive oxygen species generation, Th2 differentiation, production of interleukin (IL)-4 and IL-13 that activates B cells to differentiate into plasma cells producing allergen-specific IgE, promotes Th17 differentiation, Foxp3 expression	(312, 313)

3-MC, 3-methylcholanthrene; TCDD, 2,3,7,8-tetraclorodibenzo-p-dioxina; FICZ, 6-formylindolo[3,2-b]carbazole; KYN, kynurenine; I3C, indole-3-carbino; DIM, diindolylmethane; NOR, norisoboldine; BaP, benzo[a]pyrene, PAHs: polycyclic aromatic hydrocarbons.
